# Effect of Black Soldier Fly (*Hermetia illucens* L.) Fat on Health and Productivity Performance of Dairy Cows

**DOI:** 10.3390/ani12162118

**Published:** 2022-08-18

**Authors:** Roman V. Nekrasov, Gennady A. Ivanov, Magomed G. Chabaev, Aloyna A. Zelenchenkova, Nadezhda V. Bogolyubova, Daria A. Nikanova, Alexander A. Sermyagin, Semen O. Bibikov, Sergey O. Shapovalov

**Affiliations:** 1 L.K. Ernst Federal Research Center for Animal Husbandry, Dubrovitsy, 142132 Podolsk, Russia; 2 NordTechSad LLC, 164900 Novodvinsk, Russia; 3 Cherkizovo Research and Testing Center LLC, 107143 Moscow, Russia

**Keywords:** alternative feed, black soldier fly (*Hermetia illucens* L.), dairy cows, fatty acids, lauric acid, milk content, resistance of animals, rumen content, toxicity

## Abstract

**Simple Summary:**

What is interesting in terms of unlocking the potential of insects is their fuller and more versatile use. Insect larvae contain large amounts of protein and fat. The fat of *Hermetia illucens* larvae is primarily rich in lauric acid. Lauric acid represents a promising nutritional strategy to improve animal health and productivity. The experimental data we obtained indicate the possibility of using the insect fat for the nutrition of milking cows. The milk production and milk quality of the cows were improved by optimising digestive processes and improving body defenses. The use of fat of *Hermetia illucens* larvae can serve as an alternative to traditionally used fats of animal and plant origin, as well as preparations of biologically active substances, which will provide a sustainable resource for the diets of agricultural animals in the future.

**Abstract:**

Due to the intensive development of technologies for obtaining protein, energy feed and biologically active supplements from insects, the feasibility and effectiveness of introducing these products into the rations of farm animals require further study. This research aims to study the possibility and effects of feeding dairy cows fat from the larvae of the black soldier fly (BSFLF). The composition and properties of the BSFLF have been studied. The research of the fatty acid composition of BSFLF showed a high content of saturated fatty acids, including 58.9% lauric acid. The experiment was performed on black-and-white cows at the beginning of lactation (control, D0 (n = 12) vs. experimental D10 (n = 12) and D100 (n = 12) groups, 10 and 100 g/head/day BSFLF, respectively. There was no negative effect of BSFLF feeding on the process of feed digestion. The pH of the rumen content decreased (6.80 ± 0.07 & 6.85 ± 0.09 vs. 7.16 ± 0.06, *p* < 0.05), with an increase in the number of infusoria (0.27 ± 0.03&0.37 ± 0.09 vs. 0.18 ± 0.03 g/100 mL, *p* = 0.16); there was an increase in the concentration of VFA in the rumen content of animals of the experimental groups by 2.1 (*p* < 0.05) and 3.81 (*p* < 0.01) (8.66 ± 0.46 & 10.37 ± 0.42 vs. 6.56 ± 0.29) mmol/100 mL. The average daily milk yield of Group D10 cows over the experimental period (d17–d177) was slightly higher than the control (by 4.9%, *p* = 0.24 vs. Group D0). At the same time, Group D100 cows showed a significant increase in natural-fat milk compared to controls (by 8.0%, *p* < 0.05 vs. Group D0) over the same experiment period. Analysis of the fatty acid composition of the milk of the experimental animals showed some changes in the fatty acid composition of milk under the influence of BSFLF feeding; these changes were especially noticeable in Group D10. Thus, it was found that feeding dairy cows BSFLF at different dosages leads to better indicators of pre-gastric digestion and productivity.

## 1. Introduction

The nutritional value of insects, combined with their efficiency in converting food waste and their low water requirements, makes them a more informed choice for animal products [1]. Insects contain enough protein, fat, and minerals to contribute to global health and food security through both direct human consumption and indirect use in animal feed [2,3].

Until recently, in the EU, authorization for the use of insects in farm animal feed was limited to seven insect species (Commission Regulation (EU) 2017/893) [4], including 3 cricket species (*Acheta domesticus*, *Gryllodes sigillatus*, *Gryllus assimilis*), 2 mealworm species (*Tenebrio molitor*, *Alphitobius diaperinus*) and 2 fly species (*Hermetia illucens*, *Musca domestica*). However, gradually, the use of insect species is expanding. Since domestic silkworm feeds only on mulberry leaves (*Morus alba* and *Morus nigra*), there is no risk of contamination by food of animal origin that is not permitted for feeding insects. Silkworms (*Bombyx mori*) have been added to the list of approved insect species for the production of processed animal protein for animal feed (Commission Regulation (EU) 2021/1925) [5].

The yellow mealworm (*Tenebrio molitor*) [6,7] black soldier (*Hermetia illucens*) [8,9], housefly (*Musca domestica*) [10,11] are the most studied to date. *Hermetia illucens* has been widely studied and used worldwide in recent years [12]. Dried larvae, as well as skimmed and skimmed protein concentrates from the larvae of this fly species, have been well studied and proven to be effective in feeding pigs [13,14], poultry [15,16], fish [17,18].

The unique amino acid composition of protein, the fatty acid composition of insect lipids, and the biologically active substances they contain, allow for us to consider the biomass of black soldier fly larvae as the basis of functional nutrition [19,20,21].

Thus, larvae, pre-pupae, pupae, and adult flies of *Hermetia illucens* contain eumelanin-type pigments, which are present not only in the cuticle but also in the insect body in complex with lipids. In larvae, this is mainly lauric acid, which is associated with a melanin–chitosan complex with a wide spectrum of antibacterial activity against *Aspergillus niger*, *Candida albicans, Salmonella* and *Staphylococcus aureus* [22,23,24].

In general, larvae contain a high fraction of lipid mass (up to 578 g/kg); their amount depends on the substrate on which larvae are grown [25,26], and their composition is characterized by low amounts of monounsaturated (MUFA) and polyunsaturated (PUFA) fatty acids, and high amounts of saturated (SFA): lauric, 47.06; palmitic, 15.17; myristic, 10.48; stearic, 3.47 margarine, 0.21%. The spectrum of acids is very similar to that of palm kernel fat and coconut oil [27].

The results of Choi et al. [28] show that hexandic acid isolated from *Hermetia illucens* larvae has antibacterial properties and effectively inhibits the growth/proliferation of pathogenic bacteria (*Staphylococcus aureus*, MRSA, *Klebsiella pneumoniae* and *Shigella dysenteriae*). In this regard, the fat of larvae can be considered not only as a highly nutritious feed ingredient, but also as a potential means of functional nutrition, used for the prevention and treatment of animals. A study by Saviane et al. [29] confirms that fat from *Hermetia illucens* larvae and other active oil compounds extracted by hot pressing could be effective antimicrobial agents against various pathogenic bacteria.

It should be noted that the ban on feeding agricultural species does not apply to whole insects or to fats derived from insects [30]. At the same time, studies on the efficiency of using lipids from *Hermetia illucens larvae* in animal feed, especially ruminants, are still insufficient. The research aim is to study the possibility and effects of feeding dairy cows fat from the larvae of the black soldier fly (BSFLF).

## 2. Materials and Methods

### 2.1. Insect Fat

Black soldier fly larvae (BSFL) were obtained from NordTechSad LLC (Russia). BSFL were reared on a grain mixture. To obtain BSFLF, dried BSFLs were subjected to cold mechanical pressing (Maslyachok PSHU-4, Russia), followed by settling and bottling. The upper light fat fraction, free of impurities, was used for the study.

Lipid fraction of larvae from *Hermetia illucens* was analysed in Cherkizovo Research and Testing Center LLC (Russia): mass fraction of moisture and volatiles (ISO 662-80), mass fraction of crude fat for natural moisture (AOCS Am 5-04 rapid determination of oil/fat using high-temperature solvent extraction), acid number (ISO 660-83), peroxide number (ISO 27107-2016), mass fraction of crude protein (ISO 3960-2013), mass fraction of nitrogen (ISO 5983-2-2016), gross energy (ISO 9831-2017) [31]. The fatty acid composition of fat from larvae (ISO 12966-4) was studied.

KEMIN laboratory (Russia) used the following research methods: LB-IV-20/121-E study of oxidative stability of fat (Rancimat method); LB-IV-20/098-E determination of α, β, γ and δ-tocopherols in feed and raw material for feed production by HPLC. Relative systematic error of determination was ±10%.

### 2.2. Toxicity BSFLF

Toxicity of BSFLF was studied in the microbiology laboratory of the L.K. Ernst Federal Research Center for Animal Husbandry [32] at various component concentrations (from 0.0312 to 16 mg/mL rumen content), corresponding to (calculated) existing experimental conditions and a promising range of feeding doses, from 1 to 500 g/head/day, including the tested doses used in the experiment—10 and 100 g/head/day.

### 2.3. Animals and Housing

The experiment was conducted at the L.K. Ernst Federal Research Center for Animal Husbandry (FRCAH), located in Podolsk, Russia.

Thirty-six lactating, multiple-breeding black-motley cows, body weight (BW) 590 ± 4 kg, body fatness (BCS, according to Wildman et al. [33]) 3.15 ± 0.04, milk yield 39.1 ± 1.5 kg and days in milk (DIM) 63 ± 2 days, were identified for this experiment. Preliminary cows were ranked in descending order by DIM, milk yield, BW and BCS and assigned to control (D0; n = 12) and experimental (D10; n = 12; and D100; n = 12) groups to receive 10 and 100 g/head/day fat, respectively, from black soldier fly larvae (NordTechSad LLC, Russia) in addition to the diet (D). This allocation procedure was adopted to ensure that all experimental groups had the same DIM, milk yield, BW and BCS at d 0.

Cows were kept in individual stalls with unrestricted access to water and a total mixed ration (TMR; 1.5 m linear cow space per cow) during the experimental period. Cows were milked twice a day in a parallel milking system (05-00 and 16-00 h).

### 2.4. Experimental Diets of Cows

The diet for dairy cows met the energy and nutritional value requirements for high-yielding animals (32–36 kg milk yield) [34,35]. Feeding rations were calculated using the software complex KormOptimaExpert (Version 2016, KormoResurs LLC, Russia).

Dry matter (DM) and crude ash (CA) (ISO 6496-83 and ISO 5984, respectively) were determined at the Department of Animal Physiology and Biochemistry, FRCAH. Nitrogen content was analysed according to ISO 5983-2-2016, from which crude protein (CP) content was calculated according to the formula (nitrogen × 6.25). Crude fiber was analysed according to ISO 6865-2015, crude fat according to ISO 6492:1999, calcium (Ca) ISO 6490-1:1985, phosphorus (P) ISO 6491-2016; ME was calculated [36]. The amount used for cow feed was determined based on the hypothesis of a possible positive BSFLF effect on animal digestion, toxicity data, and manufacturer’s recommendations (NordTechSad LLC, Novodvinsk, Russia) (10 and 100 g/head/day of fat).

BSFLF was absent in the control (D0) diet, and was fed in amounts of 10 and 100 g/head/day in the corresponding experimental diets D10 and D100, respectively (Table 1).

The total duration of the research period was 176 days, including a preliminary period of inoculation to the new feed for 17 days, feeding the lipid fraction according to the experiment scheme for 90 days, and further accounting for the aftereffect for 69 days. From day 1 to day 17 of the experiment, the cows in the experimental groups were accustomed to the new fodder, given 1/2 dose of fat, according to the general feeding scheme. From day 18 to day 107 of the experiment, fat was fed at 10 and 100 g/head/day. The BSFLF was given to the cows individually by hand; TMR was given once during the morning feeding by mixing it with the main feed.

Before feeding, the fat was first chopped in an electric meat grinder (Moulinex ME856D32, France) with a sieve size of 5 mm, then cooled in a refrigerator at 4 ± 2 °C for 24 h, after which it was placed in individual plastic containers of 10 and 100 g.

### 2.5. Milk Performance and Analyses in Milk

The average daily milk yield was calculated from the results of control milkings before the experiment (d 0), on days 17, 42, 67, 92, 117 of the main experimental period (d 17; d 42; d 67; d 92; d 117), on days 147 and 177 (d 147; d 177) during the study of the effects of fat.

Average daily milk samples on these days were taken from each cow (n = 36) and an infraspectrometric analyzer (in the Department of Population Genetics and Genetic Bases of Animal Breeding, FRCAH) CombiFoss 7 DCC (FOSS, Denmark) determined the following: fat, protein (pure and crude), casein, lactose, dry matter (SNF and TS), urea, citric acid, fatty acid (FA) profile analysis (C14:0 (myristic), C16:0 (palmitic), C18:0 (stearic), C18: 1 (oleic), sum of saturated FA, monounsaturated FA, polyunsaturated FA, short-chain FA, medium-chain FA, long-chain FA, and trans-FA isomers), free fatty acids, lower freezing point, pH, ketosis test, total somatic cell count (SCC) by standard methods (ISO 9622/IDF 141:2013; AOAC 972. 16). The MilkoScan™ 7 RM is CE-marked for standards and validation.

Based on the results, the mean values for each indicator were calculated for the animal group before the start of the experiment and at subsequent sampling control points.

### 2.6. Blood and Rumen Fluid Sampling and Analyses

Blood samples from the jugular vein were collected at the end of the main experimental fat-feeding period (d 117) at 09-00 h. Blood was collected in vacuum containers (Vacuette, Greiner bio-one, Kremsmünster, Austria), with a blood-clotting activator to determine (in the Department of Physiology and Biochemistry of Farm Animals of L.K. Ernst Federal Research Center for Animal Husbandry) calcium (Ca), phosphorus (P), magnesium (Mg), aspartate aminotransferase (AST), alanine aminotransferase (ALT), alkaline phosphatase (ALP), total bilirubin (TBIL), creatinine (CREA), cholesterol (CHOL), glucose (GLU), triglycerides (TRIG), total protein (TP), albumin (ALB) and urea (UREA). Blood samples were stored on ice immediately after withdrawal and centrifuged after 30 min. All samples were centrifuged at 4 °C at 6000 rpm for 5 min (centrifuge, Hettich GmbH & Co KG, Germany). Samples were subsequently stored at −20 °C until analysis. A ChemWell 2910 automatic biochemical and immunoassay analyzer (Awareness Technology, USA) was used for the analysis. The total amount of water-soluble antioxidants (TAWSA) in blood serum of experimental animals was determined by amperometric method (chromatograph, TsvetYauza 01-AA, SPA Shimavtomatika PLC, Russia).

Then, blood was drawn from each cow into vacuum containers (Vacuette, Greiner bio-one, Kremsmünster, Austria), with K3 EDTA to determine erythrocytes (RDC), leukocytes (WBC), hemoglobin (HGB), hematocrit (HCT) and transported to the laboratory within two hours. A hematological analyzer ABC VET analyzer (Horiba ABZ, France) with Uni-Gem reagent kits (ReaMed, Russia) was used for analysis.

Rumen fluid was taken on d 117 of the experiment 3 h after the morning milking using a food probe in three animals from each group, typical of the average milk productivity of each group during the experiment. Rumen content was examined using standard methods (in the Department of Physiology and Biochemistry of Farm Animals of the L.K. Ernst Federal Research Center for Animal Husbandry): pH, total volatile fatty acids—by steam distillation in Markgam apparatus; ammonia nitrogen—by Conway microdiffusion method; amylolytic activity—photometric method; oxidation by Krylova and Lyaskovskaya.

### 2.7. Bacterial Strains and Culture Conditions

In the Laboratory of Microbiology (FRCAH), in selected blood samples, the indices of nonspecific resistance of experimental animals, the bactericidal, lysozyme activity of serum and phagocytic activity of blood cells, were determined using conventional methods.

#### 2.7.1. Phagocytosis Assay

*E. coli* culture (0.5 mL) was added to 0.5 mL of blood and incubated on shaker at 37 °C for 30 min. The mixture’s sediment was smeared, fixed with 96% methanol, stained with Romanowsky–Giemsa method and viewed under microscope (90×). *E. coli*-engulfed neutrophils were counted as positive cells. We analysed 100 neutrophils per slide. The following parameters were determined:

Phagocytic activity (PA) = (Number of neutrophils involved in phagocytosis/all neutrophils) × 100%;

Phagocytic index (PI) = Number of E. coli cells ingested/100 active neutrophils;

Phagocytic amount (PAM) = Number of phagocytosed bacteria cells/all neutrophils.

#### 2.7.2. Lysozyme Activity Assay

Lysozyme was measured by turbidimetric method in a spectrophotometer UNICO-2100 (united products and instruments, Ins. USA) at OD540. The following parameters were determined: lysozyme activity of blood serum (LA), concentration of serum lysozyme (lysozyme, µg/mL), activity unit (AU) per 1 mg protein (AU/TP).

Lysozyme activity (LA) of a blood serum was calculated using the following formula:

%LA = ((ΔDo) × 100/Do1) − ((ΔDk) × 100/Dk1).

Do is the difference in the optical density of the prototype.

Dk is the difference in the optical density of the control.

Do1 is the immediate optical density of the prototype immediately.

Dk1 is the optical density of the control.

Due to variations in the protein content in the blood serum of animals, the level of lysozyme activity was converted and expressed in arbitrary units of activity per 1 mg of protein (activity units per 1 mg of TP or AU/TP).

#### 2.7.3. Bactericidal Activity of Blood Serum

Bactericidal activity (BA) of blood serum was measured by turbidimetnic method in a spectrophotometer UNICO-2100 (united products and instruments, Ins. USA) at OD540.

Percentage of BA was calculated from the following formula:

%BA = ((Dk −Do)/Dk) × 100.

Dk is optical density of control.

Do is optical density of experimental sample.

### 2.8. Statistical Analyses

Statistical analyses of the data were performed with STATISTICA software (version 13RU, StatSoft, Inc., 2011) using a general linear model. Each group was considered as an experimental unit in measurements of milk performance, while individual cows were used as an experimental unit for analyzing nutrient blood and rumen characteristics. Quantitative data are presented as arithmetic mean (M) and mean square error (MSE). The relationship between the factor under study and the studied parameters was detecting using a sample of animals, one-factor analysis of variance (ANOVA) and Dunnett’s criterion. Statistical differences were considered highly significant at *p* < 0.01, significant at *p* < 0.05 and a tendency at 0.05 < *p* ≤ 0.1 ^a^—*p* < 0.05; ^b^—*p* < 0.01; ^c^—*p* < 0.001; ^d^—*p* ≤ 0.10.

### 2.9. Ethical Approval

Ethical approval for the study was provided by the bioethical commission of the L.K. Ernst Federal Research Center for Animal Husbandry (protocol #2020-01/1, dated 14 January 2020).

## 3. Results

### 3.1. Chemical Composition and Characteristic BSFLF

**Figure 1:**
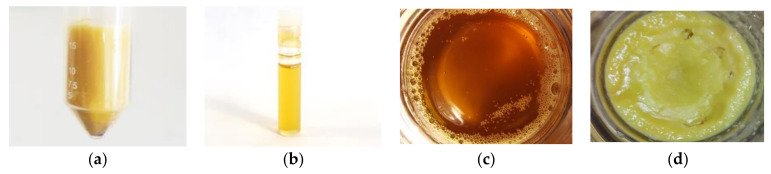
Fat of Black soldier fly larvae: (**a**) native; (**b**) after centrifugation; (**c**) liquid (t = 40 °C); (**d**) solid (t = 20 °C).

The chemical composition and some properties of the lipid fraction of *Hermetia illucens* larvae were studied. Almost no impurities were found in the sample; the fat MD was 99.9%. In terms of energy value (38.4 MJ), this corresponded to fats of animal origin (Table 2, Figure 1).

**Figure 2:**
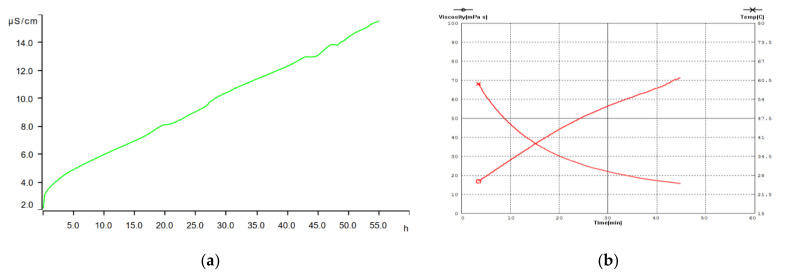
BSFLF properties: (**a**) oxidative stability curve of the BSFLF sample; (**b**) dependence of viscosity of the BSFLF sample on temperature and time.

Figure 2 shows the oxidative stability of the samples tested with the Rancimat test. The induction point shows the deterioration of the sample under test conditions. The decrease in product viscosity was established as depending on temperature and time (Table 3, Figure 2).

**Figure 3:**
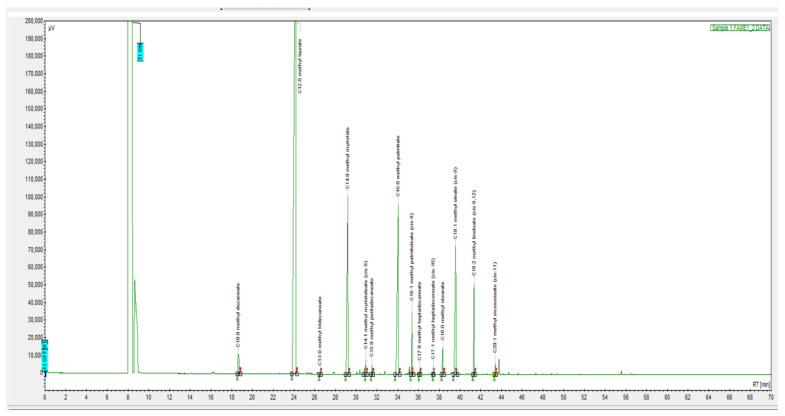
Chromatogram of fat of *Hermetia illucens* larvae.

The study of the fatty acid composition (Table 4, Figure 3) indicates the presence of a high saturated fatty acid content in BSFLF: lauric acid—58.9; myristic acid—11.1; palmitic acid—12.7; stearic acid—1.2%, the proportion of unsaturated was: palmitoleic acid—2.2; oleic acid—7.4; linoleic acid—3.5%.

### 3.2. Toxicity BSFLF

No BSFLF toxicity was detected (Table 5). All concentrations of the tested sample had no negative effect on the motility, movement, and cell shape of *T. pyriformis infusoria*.

### 3.3. Milk Productivity of Dairy Cows

**Figure 4:**
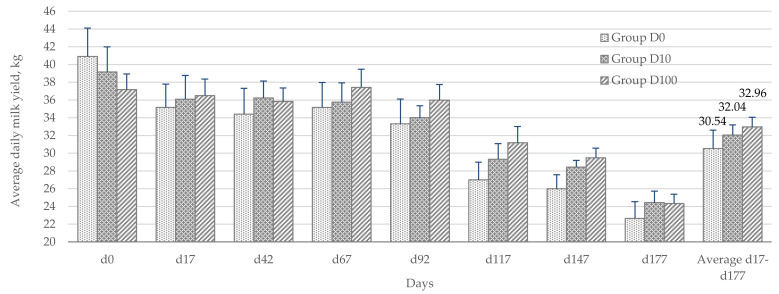
Milk yield of cows (n = 12) of natural fat, kg.

The average daily milk yield of Group D10 cows over the experimental period (d17-d177) was slightly higher than the control (by 4.9%, *p* = 0.24 vs. Group D0). Group D100 cows showed a significant increase in natural-fat milk compared to controls (by 8.0%, *p* < 0.05 vs. Group D0) over the same experiment period (Figure 4).

### 3.4. Milk Content

There were no significant differences in the composition and quality of milk. At the same time, milk from Group D10&D100 cows was characterized by a higher dry matter content (*p* < 0.1, D10 vs. D0). The ratio of fat and protein content in milk of experimental groups was also more optimal—1.09-1.1 vs. 1.05 in the control. There was a significant decrease in acetone content and, to a lesser extent, BHB content, in the milk of experimental groups.

An analysis of the fatty acid composition of the milk of the experimental animals showed some changes in the fatty acid composition of milk under the influence of BSFLF feeding; these changes were especially evident in Group D10. Saturated fatty acids (SFA) were predominant in milk of all groups. It should be noted that the total proportion of «desirable» unsaturated FA (MUFA, PUFA) in milk samples in Groups D10&D100 was 1.12–1.22 vs. 1.07% in the control (Table 6).

### 3.5. Parameters of Rumen Contents of Cows

Feeding animals additional BSFLF resulted in a decrease in rumen content pH (*p* < 0.05 in D10&D100 groups compared to D0, within the normal range of 6.5–7.0 units). This occurred against the background of the more favorable conditions of better cleavage of fiber to volatile fatty acids (VFA) (*p* < 0.05 vs. control D0) by microorganisms in the rumen of experimental groups D10&D100. The increase in total microbial mass (by 0.05–0.19 g/100 mL, *p* > 0.05) occurred in animals in experimental groups due to the growth of infusoria. The positive effect of BSFLF was also evidenced by the increased amylolytic activity in the rumen of D10&D100 cow groups, by 3.11 (*p* < 0.05) and 1.94 U/mL (*p* > 0.05) vs. control. There was a decrease in ammonia formation in the rumen of animals of the D10&D100 (at *p* < 0.05, D100 vs. D0), which also indicates that BSFLF supplementation contributed to the more efficient use of feed nitrogen (Table 7).

### 3.6. Blood Parameters of Experimental Animals

The use of BSFLF in the diets of highly productive cows did not lead to significant changes in the biochemical blood profile (Table 8). The stabilization of blood serum TP in D10&D100 groups of animals at the 90.32–90.70 g/L (*p* > 0.05 vs. D0) level was accompanied by corrections to the protein index (ALB/GLB ratio) by 0.06 and 0.09 units (*p* > 0.05), while these parameters in the D10&D100 groups of animals were within the norms (TP, 70–92 g/L; ALB/GLB, 0.6–1.0), vs. Group D0. There was an increase in the serum UREA content in the blood of the experimental groups of cows (within the reference interval, UREA, 2.35–7.06 mmol/L): by 1.62 mmol/l (*p* < 0.001) in the Group D10 and by 0.33 mmol/l (*p* = 0.35) in Group D100. There was a decrease (*p* < 0.05) in CHOL concentration in the blood of cows from Group D100 (which consumed 100 g/cow/day of BSFLF), against the background of an increase in TBIL (within the physiologically established reference parameters; CHOL, 2.35–8.30; mmol/L; TBIL, 1.16–8.18 µmol/L) in the blood of Group D10 (*p* = 0.09) and Group D100 (*p* < 0.05). Morphological blood parameters in all groups were within the physiological norm and there were no differences between the groups.

Cows in experimental groups fed BSFLF showed an increase in serum lysozyme activity in the D10 (*p* < 0.05) and D100 (*p* = 0.08) experimental groups, respectively, compared to the control D0, which corresponded to the increase in lysozyme content and its activity in 1 mg protein in the Group D10 (*p* < 0.05) and a tendency towards an increase in the Group D100 (*p* = 0.013; *p* = 0.08). Feeding BSFLF to cows resulted in an increase of bactericidal activity in blood serum in Group D100 (*p* < 0.05) compared with the control (Table 9).

## 4. Discussion

Research on new ingredients from insects is particularly relevant due to the growing shortage of animal feed [1,37,38]. The use of not only protein but also fat from insect larvae is of interest because their fat content is comparable with the protein content. Barragan-Fonseca et al. [39] found that the protein content of larvae can be regulated within narrow limits, while the crude fat content of larvae strongly depends on the concentration of nutrients and the density of larvae breeding.

Compared to the use of insect protein, not enough research has been conducted on the use of fat [40]. Therefore, due to the quality and high fat content of insect larvae, there is a need for increased experimentation to study their application in animal feed rations, as a replacement for traditional dietary energy resources and functional supplements.

In our research, the nutritional value, properties and fatty acid composition of BSFLF were investigated in trials. The energy value of the studied product was high (38.4 MJ), comparable to that of animal fats and higher than that of plant lipids, suggesting that BSFLF is an important source of energy for animals.

Based on the oxidation parameters that were obtained, the tested fat sample corresponded to a fresh product with high oxidation degree characteristics, which is an important characteristic when storing and using the fat. The estimated storage time at 20 °C was more than 512 days (according to the theoretical data of the accelerated oxidation test—Rancimat test, Figure 2), indicating the high stability of the studied fat sample. The level of acid number (1.6 mg KOH/g fat) was quite low, indicating no hydrolytic deterioration of BSFLF.

A number of animal and plant lipids contain natural antioxidants. The most active are tocopherols (Vitamin E). In vegetable oils, they contain from 0.01 to 0.28. In our studies, the total content of tocopherols was found to be 75 mg/kg (or 0.0075%). Melanin is among the most powerful natural antioxidants. The presence of melanin may also contribute to long-term storage [41]. In earlier studies, we found that, in the suspension prepared for testing from BSFL, the concentration of melanin was 1.2 mg/mL [22]. There are also reports on the anti-stress effect of melanin [42]: when it was added to the diet of animals (0.1 mg/kg) no cases of disease were observed; it was shown [43] that, in stressed animals, melanin promotes the normalization of proteinase-inhibitory potential and prevents the development of pancreatic cytolytic syndrome. In our experiment, serum TAWSA in high-yielding cows at 4–5 months of lactation was 7.9–17.3 mg/g (Table 8). The highest value for this index was found in Group D100—12.59 mg/g blood serum (*p* = 0.15) compared with 10.96 mg/g in the control, indicating the increased antioxidant properties of the blood serum of experimental animals under the influence of BSFLF feeding.

The production of lipid supplements is challenging because many fats (mostly of animal origin) are in solid form at room temperature, and so must be melted and processed for storage, transport and mixing. One of the main indicators for emulsions is the effective viscosity, which characterises the flow behaviour of the fluid. With increasing temperature, the viscosity of BSFLF decreases from 71.15 (25 °C) to 16.47 (60 °C) cPs (Table 3). These data are broadly comparable to Maltseva [44], those in which the rheological properties of larval fat at different temperatures (20 to 95 °C) varied from 1500 to 5.7 cPs. Ruban et al. [45] showed that the introduction of BSFLF to a solution, followed by mechanical dispersion (preparation of emulsions), reduced the consistency of K solutions by half, regardless of lecithin concentration. The data we obtained supplement the already known data and can be used in the process of fat extraction from larval biomass, as well as being taken into account when storing the fat, adding it to the diet, and the physiological justification of feeding animals this diet.

The inclusion of fat additives of various origins (animal, vegetable, etc.) in ruminant diets is a primary way of increasing the feed energy concentration. In normal diets of dairy cows, fat is contained in small amounts (2–3%), but when cows have a negative energy balance immediately after calving and during peak lactation, this deficit can be addressed by feeding more fat, up to 6% of the dry matter content of the diet, without increasing the feed quantity.

Calcium saponified fats [46], inert fats, medium-chain triglycerides [47,48], free fatty acids [49] and others are successfully used in ruminant feeding. According to Sosa and Fogliano [50], insect lipids are classified based on the fatty acid composition between plant oils and animal fats. Many researchers have found that BSFLF is similar to coconut oil and palm oil in its qualitative and quantitative fatty acid composition and is solid at room temperature, which makes it a promising alternative fat source for use in animal feed [51]. According to Kierończyk et al. [52] fats from insects have higher concentrations of monounsaturated fatty acids (MUFA) and lower polyunsaturated fatty acids (PUFA) compared to other oils. Fats from insects can be considered biologically active compounds that modify the molecular structure at the mRNA level. The fat sample produced by LLC NordTechSad (Russia) was found to have the highest saturated fatty acid (SFA) content, at 86.0%, including a lauric acid content of 58.9%. According to Alifian et al. [53], the SFA content of BSFLF averaged at 74%, and the lauric acid content 40.5–46.7%; according to Ewald et al. [24], the average was 60.5% SFA, and 13.4–52.1% when including lauric acid. Significant differences in fatty acid composition in the studies are related, firstly, to the diet of insect larvae and growing technology, and, secondly, to the technical conditions for obtaining fat from larvae. We note that the high content of saturated acids (SFA) and lauric acid in our sample compared with previous studies [24,53] is associated with larval rearing on cereal raw materials. High levels of SFA in BSF larvae and prepupae have also been shown in Renna et al. [54]—80.3% SFA when larvae were reared on vegetable byproducts substrate, Meneguz et al. [55]—81.9% SFA on fruit waste, Spranghers et al. [56]—82.8% SFA in prepupae reared on vegetable waste, Jucker et al. [57]—86.0% SFA on fruit, Danieli et al. [58]—81.05–86.89% SFA on various combinations of wheat middlings, wheat straw, ground barley and dehydrated alfalfa.

An important factor in the use of fats in ruminant diets is their security, i.e., when higher energy levels are provided by the introduction of fat additives without harming the rumen microflora, while reducing the risk of acidosis. Such fats are not broken down in the rumen, but enter the rumen in an acidic environment, undergo hydrolysis and are digested in the small intestine. As mentioned previously, lauric acid has antimicrobial activity [59], which may affect the improvements in animal body defenses when lauric acid is used in the diet [28]. The BSFLF study showed no toxicity of different doses (0.0312–16.0 mg/mL) on the test culture of infusoria (Table 5). In the experiment on dairy cows, there was even an increase (*p* = 0.16) in total microbial mass due to infusoria growth (Table 7). The doses of BSFLF we studied, especially 100 g/head/day, had no negative effect on rumen metabolism. There was an increase in amylolytic activity (*p* < 0.05), a better breakdown of fibre to volatile fatty acids (*p* < 0.05), and a decrease in the pH of the rumen contents (*p* < 0.05).

Fats have a nitrogen-saving property, based on a reduction in the use of amino acids, which helps to meet the body’s energy requirements and direct them to the synthesis of microbial mass proteins. We also found that feeding BSFLF to dairy cows led to a decrease in ammonia formation in the rumen of the experimental animals (*p* < 0.05). There was an increase in serum urea in group D10 cows of 1.62 mmol/l (*p* < 0.001) and, in group 3, by 0.33 mmol/l (*p* = 0.35), which could indicate the improved protein use by the animals, together with a reduction in total blood protein (Table 8).

The fatty acid composition of milk lipids determines the sensory properties of milk as well as its properties during storage, particularly the intensity of the lipolysis and oxidation processes. The vast majority of saturated fatty acids enter the bloodstream via the lymph and are transported to the mammary gland, where they participate in milk fat synthesis. In contrast to LCFAs, which are absorbed through the lymphatic system as chylomicrons, MCFAs are absorbed through the hepatic portal vein [47].

Sun et al. [48] evaluated unprotected lipid supplementation (400 g per head per day) with different ratios of MCFA to LCFA (20:80; 40:60; 60:40). The percentage of milk fat and total SMCFA concentration in milk fat tended to linearly increase, and the proportion of total milk solids linearly increased with increasing SMCFA to LCFA ratio in supplements, while milk fat yield did not change. Increasing the SMCFA to LCFA ratio in the diet could potentially improve milk fat synthesis. In our studies (Table 6), feeding BSFLF resulted in a higher milk fat content in the milk of D10&D100 cows (*p* = 0.16), with a better fat to protein ratio of 1.09–1.1 in the experimental groups compared to 1.05 in the control, which is important for controlling herd acidosis, especially early in lactation, in diets with a high proportion of concentrated feed. Medium-chain fatty acids are mainly formed by de novo synthesis from substrates such as acetate and butyrate in the mammary gland [60], which is consistent with the evidence of increased volatile fatty acid content in the rumen contents of D10&D100 cows. Overall, BSFLF feeding resulted in a marked increase in SCFA&MCFA in the fatty acid composition of milk fat.

Ketone bodies are indicators for the diagnosis of an impaired metabolism. Feeding diets supplemented with BSFLF resulted in lower acetone and BHB content (*p* < 0.05) in the milk of D10&D100 cows, which improved lipid metabolism and enhanced the energy and plastic requirements of the body. We primarily attribute this improvement to the positive effects on the rumen microflora, as quantitatively feeding BSFLF was not high relative to the animal’s energy requirements.

According to a number of authors, the cholesterol content in the blood of healthy cows directly correlates with milk productivity. Cholesterol is mostly produced by liver cells and is located in the vascular wall throughout the body. The lipoprotein has a building hormone-producing function, is involved in the absorption of vitamin D, improves digestion and participates in the serotonin receptor system. A decrease (*p* < 0.05, Table 8) in cholesterol concentration in the blood of D100 cows should be noted, but the index was normal against the background of rather high values in the control and D10 groups, which may indicate the effect of feeding the factor under study on liver function. This is also proved by the increase (within physiological limits) in total bilirubin in the blood of animals in the D10 group (*p* = 0.09) and D100 group (*p* < 0.05) vs. control.

Formed as a result of hemoglobin and myoglobin decomposition, this pigment is excreted with bile, which acts as the main cellular antioxidant—a substance that binds free radicals, slows down oxidation processes, and helps to restore the destroyed red blood cells. Experiments in rat [61], poultry [62,63] models have also shown that lauric acid administration reduces serum total cholesterol and high-density lipoprotein (HDL) levels, which may be associated with the high MCFA levels in BSFLF [64].

*Hermetia* larvae have long been used in Europe and America as a medical resource and medicinal insect to treat skin injuries such as burns and wound healing [28]. These effects are related, among others, to the FA composition of BSFLF. As noted earlier, lauric and other acids have strong antimicrobial activity. A study [65] investigated the effects of calcium saponified lauric acid (C12-Ca) on growth performance and intestinal health in piglets after weaning. In the duodenum and ileum, the administration of C12-Ca provided higher total antioxidant capacity and lower malonic dialdehyde levels (*p* < 0.001). C12-Ca improved ileal villous height and width (*p* < 0.001). Another supportive study for the high antibacterial potency of LA was conducted with different types of Gram-positive bacteria, and further demonstrated that unsaturated fatty acids with 18 carbon long chains—oleic acid (C18:1), linoleic (C18:2), and linolenic acid (C18:3)—have potent antibacterial activities as well [66].

Research by Erickson et al. [67], Liu et al. [21] emphasise BSFL’s antibacterial activity against *Salmonella* spp. and *Escherichia coli,* suggesting that larvae could be used in organic waste treatment and drug therapy.

Our results show that BSFLF administration not only improved the digestive function and health of dairy cows, but also leads to improved protective functions. For example, BSFLF feeding was associated with a slight decrease in somatic cell count in the milk of D10&D100 animals (*p* = 0.37, Table 8). Previously, in young cattle, we showed a significant effect of feeding melanin protein-energy supplement of *Hermetia illucens* larvae on non-specific immunity and intestinal microbiocenosis composition in experimental animals [68]. There is evidence of increased immunity in animals when feeding them various components of insect larvae [40]. According to Yu et al. [69], feeding *Hermetia illucens* larvae can enhance mucosal immune homeostasis in pigs by altering the bacterial composition of intestinal contents and their metabolites. The use of *Hermetia illucens* larvae increases the frequency of CD4+ lymphocytes in broiler chickens, indicating their positive effect on immune homeostasis.

We also attribute the obtained effects to the composition of FAs and the action of lauric and other medium-chain acids contained in BSFLF. The lauric and other acids in BSFLF exhibit significant antibacterial activity due to their ability to disorder lipid membranes of microorganisms, making BSFLF suitable not only for animal nutrition, but also as a functional food supplement for the treatment and prevention of certain diseases [70].

Thus, feeding BSFLF improved the cows’ body defenses as well as their rumen digestion and metabolism. The results included an improvement in milk quality and animal health. Feeding BSFLF had a positive effect on the milk productivity of the cows (Figure 4). The highest efficiency was shown when using 100 g/head/day of BSFLF (32.96 vs. 30.54 kg of milk, Figure 4). Therefore, while the use of insect larval fat is predetermined by its composition and properties, its full targeted use is still hampered by the lack of a complete understanding of its possible utilization. Studies on the applicability of insect fat have been conducted on broilers [57,71,72], laying hens [73], turkeys [62], rabbits [74] and fish [75]. Although BSFLF is rich primarily in lauric acid, the effect of lauric acid, as well as BSFLF on rumen bacteria is less well known [76]. Our research fills this gap to a certain extent.

## 5. Conclusions

The nutritional characteristics of BSFLF need to be further investigated, including the development and production of feed formulations for different groups of farm animals, incorporating insect biomass as an alternative to traditionally used ingredients. Our research considered only two dosages (10 and 100 g/head/day) for dairy cows, and the results are promising. The data we obtained showed that the use of BSFLF can result in improvements in the productivity, body defences and milk quality of the cows.

The BSFLF can be seen as a dietary supplement to, and a replacement for, traditional energy sources for ruminants (primarily palm oil). However, to increase the use of BSFLF in ruminant feed in larger quantities, more research on the technology to protect BSFLF in the rumen is needed.

Data on the biological effects of insect lipids derived from different species of insect larvae for widespread use in farm animal diets are slacking, and data on ruminants are even less sufficient. Therefore, research is needed to expand the evidence base for the applicability of insect larval components in the nutrition of animal species, including ruminants. Research should focus on studying the possible action mechanisms and impact of substances from *Hermetia illucens* L. on the immunity-related genes involved in fat metabolism.

Thus, the use of BSFLF could serve as an alternative to traditionally used animal and plant fats, as well as preparations of biologically active substances, which would provide a sustainable future resource for the diets of farm animals.

## Figures and Tables

**Table 1:** Composition of experimental diets.

Parameter ^1^	Diets ^2^		
	D0	D10	D100
Ingredient (kg)			
Hay	1.0	1.0	1.0
Haylage	13.0	13.0	13.0
Corn silage	15.0	15.0	15.0
Fresh brewer’s grain	4.0	4.0	4.0
Compound feed ^3^	14.5	14.5	14.5
BSFLF	-	0.01	0.10
Calculated nutrients			
Energy (MJ/kg DM)	11.18	11.18	11.29
Analysed nutrients			
DM (kg)	24.00	24.01	24.10
Crude protein (% DM)	16.21	16.20	16.14
Crude fibre (% DM)	18.54	18.53	18.46
Starch (% DM)	22.31	22.30	22.21
Sugar, (% DM)	4.99	4.98	4.97
Crude fat, (% DM)	3.91	3.95	4.31
Calcium, (% DM)	0.69	0.69	0.69
Phosphorus, (% DM)	0.52	0.52	0.51

^1^ BSFLF or black soldier fly larval fat. DM, crude protein, crude fibre, starch, sugar, crude fat, Calcium, Phosphorus are results of chemical analysis of feed samples. ^2^ D0 = 0 g (control), D10 = 10 g, D100 = 100 g levels of supplement of BSFLF in the diets. ^3^ Compound feed contained per 1 kg: wheat 155 g, barley 200 g, corn 200 g, wheat bran 100 g, soy meal 100 g, sunflower meal 100 g, rapeseed meal 100 g, monocalcium phosphate 20 g, premix 15 g, salt 10 g. Premix contained per 1 kg: vitamin A 2,200,000 IU; vitamin D_3_ 300,000 IU; vitamin E 1500 IU; vitamin B5 (pantothenic acid) 500 mg; choline chloride 40,000 mg; magnesium (Mg) 150,000 mg, sulphur (S) 100,000 mg, iron (Fe) 500 mg, manganese (Mn) 6000 mg; zinc (Zn) 6000 mg, copper (Cu) 1500 mg; iodine (I) 150 mg, cobalt (Co) 150 mg, selenium (Se) 25 mg.

**Table 2:** Composition and properties of the fat of black soldier fly larvae.

Parameter	Mean	±Δ
Mass fraction of moisture and volatile substances, %	0.02	0.002
Mass fraction of crude fat on natural moisture, %	99.98	10.0
Mass fraction of crude protein, %	None	-
Mass fraction of nitrogen, %	none	-
Gross energy, MJ/kg	38.36	0.4
Content of tocopherols, including		
alpha-tocopherol, mg/kg	40.4	4.0
sum of beta- and gamma-tocopherols, mg/kg	25.5	2.5
delta-tocopherol, mg/kg	8.1	0.8
Gross energy, MJ/kg	38.46	0.71
Acid value, mg KOH/g fat	1.6	0.2
Peroxide value, O_2_ mmol/kg	1.66	0.2
TBA value, mg/kg	0.05	0.0005
Oxidative stability OSI/Rancimat test (induction point at 100 °C; hours)	>48	-
Oxidative stability OSI/Rancimat test (conversion to 20 °C; hours)	>12,288	-

**Table 3:** Viscosity of fat as a function of temperature, cPs.

Sample	Temperature, °C				
	25	30	40	50	60
Fat of *Hermetia illucens* larvae	71.15	54.46	34.95	23.59	16.47

**Table 4:** The content of fatty acids in BSFLF (g/100 g of FAs).

Name of Fatty Acid	Mean	±Δ
Capric acid (C10:0)	1.57	0.5
Lauric acid (C12:0)	58.93	3.0
Tridecanoic acid (C13:0)	0.06	0.5
Myristic acid (C14:0)	11.11	1.17
Myristoleic acid (cis-9) (C14:1)	0.45	0.05
Pentadecanoic acid (C15:0)	0.31	0.05
Palmitic acid (C16:0)	12.68	1.46
Palmitoleic acid (cis-9) (C16:1)	2.17	0.5
Margarine acid (C17:0)	0.13	0.05
Heptadecenic acid (C17:1)	0.1	-
Stearic acid (C18:0)	1.24	0.5
Oleic acid (cis-9) (C18:1)	7.39	0.8
Linoleic acid (cis-9,12) (C18:2)	3.52	0.5
Eicosenic acid (cis-11) (C20:1)	0.34	0.05
Total, %, including	100.00	-
SFA, %	86.03	-
USFA, %	13.97	-
USFA/SFA	0.16	-
MUFA, %e	10.45	-
PUFA, %	n/a	-

SFA, saturated fatty acids; USFA, unsaturated fatty acids; MUFA, monounsaturated fatty acids; PUFA, polyunsaturated fatty acids; n/a—not analysed.

**Table 5:** Toxicity BSFLF.

Parameter	**BSFLF Concentration, mg/mL**									
	0.0312	0.0625	0.125	0.25	0.5	1.0	2.0	4.0	8.0	16.0
Toxicity level	n/d	n/d	n/d	n/d	n/d	n/d	n/d	n/d	n/d	n/d

n/d—not detected.

**Table 6:** Indicators of average composition and quality of milk for the experimental period ^1^.

Parameter	Diets ^2^			SEM	*p*-Value
	D0 n = 36	D10 n = 36	D100 n = 36		GLM
Fat content in milk, %	3.66 ± 0.11	3.72 ± 0.13	3.72 ± 0.10	0.074	0.99
Protein content in milk, %	3.47 ± 0.04	3.42 ± 0.06	3.38 ± 0.04	0.028	0.16
Lactose, %	4.67 ± 0.10	4.73 ± 0.03	4.81 ± 0.03	0.037	0.15
SNF, %	8.95 ± 0.13	8.95 ± 0.07	8.98 ± 0.05	0.051	0.92
Dry matter, %	12.45 ± 0.23	12.98 ± 0.21 ^d^	12.62 ± 0.13	0.114	0.16
Casein, %	2.72 ± 0.04	2.70 ± 0.05	2.67 ± 0.03	0.024	0.42
Acetone, mmol/l	0.06 ± 0.01	0.04 ± 0.01 ^d^	0.03 ± 0.01 ^a^	0.004	0.01
β-hydroxybutyrate, mmol/L	0.05 ± 0.01	0.03 ± 0.01	0.03 ± 0.01 ^d^	0.005	0.03
Urea, mg/100 mL	39.88 ± 1.27	42.66 ± 1.15	43.30 ± 0.97 ^a^	0.671	0.03
Freezing point	533.83 ± 1.09	531.74 ± 2.02	534.57 ± 0.83	0.816	0.60
Acidity, pH	6.56 ± 0.03	6.60 ± 0.02	6.60 ± 0.01	0.014	0.31
Fatty acids, g/100 g, including					
myristic acid	0.36 ± 0.01	0.40 ± 0.02 ^a^	0.36 ± 0.01	0.008	0.14
palmitic acid	0.98 ± 0.04	1.12 ± 0.06 ^a^	0.97 ± 0.03	0.026	0.22
stearic acid	0.32 ± 0.02	0.36 ± 0.02	0.32 ± 0.01	0.011	0.41
oleic acid	0.99 ± 0.04	1.14 ± 0.06 ^a^	1.07 ± 0.04	0.027	0.06
long chain fatty acids (LCFA)	1.17 ± 0.06	1.35 ± 0.08 ^d^	1.27 ± 0.05	0.039	0.09
medium chain (MCFA)	1.51 ± 0.05	1.70 ± 0.08 ^a^	1.51 ± 0.05	0.036	0.24
short-chain (SCFA)	0.46 ± 0.02	0.55 ± 0.03 ^b^	0.49 ± 0.02	0.013	0.02
saturated (SFA)	2.46 ± 0.10	2.82 ± 0.14 ^a^	2.51 ± 0.08	0.064	0.14
monounsaturated (MUFA)	0.94 ± 0.04	1.09 ± 0.06 ^a^	1.00 ± 0.03	0.026	0.08
polyunsaturated (PUFA)	0.12 ± 0.004	0.13 ± 0.01 ^d^	0.12 ± 0.003	0.002	0.09
trans-isomers (TFA)	0.08 ± 0.005	0.08 ± 0.01	0.07 ± 0.01	0.003	0.88
Somatic cells, thousand/cm^3^	323.80 ± 102.08	238.91 ± 39.05	258.71 ± 50.23	40.08	0.37

^1^ Means (± standard error) within a row (overall) followed by different superscript are significantly different, General Linear Model (GLM), *p* < 0.05, least significant difference test. ^2^ D0 = 0 g, D10 = 10 g, D100 = 100 g, levels of supplement BSFLF. ^a^—*p* < 0.05; ^b^—*p* < 0.01; ^d^—*p* ≤ 0.10. vs. control. For each diet, n = 36 (3 taking × 12 head).

**Table 7:** Parameters of rumen contents of experimental animals ^1^.

Parameter	Diets ^2^			SEM	*p*-Value
	D0	D10	D100		GLM
	n = 3	n = 3	n = 3		
pH	7.16 ± 0.06	6.80 ± 0.07 ^a^	6.85 ± 0.09 ^a^	0.07	0.03
VFA, mMol/100 Ml	6.56 ± 0.29	8.66 ± 0.46 ^a^	10.37 ± 0.42 ^b^	0.62	0.001
Ammonia nitrogen, mg%	16.08 ± 0.05	11.66 ± 3.47	11.74 ± 1.06 ^a^	1.41	0.31
Amylolytic activity, U/ml	13.35 ± 0.51	16.46 ± 0.59 ^a^	15.29 ± 1.37	0.68	0.13
Microorganisms, total, g/100 mL of rumen content, including	0.61 ± 0.10	0.66 ± 0.03	0.80 ± 0.17	0.07	0.53
Infusoria	0.18 ± 0.03	0.27 ± 0.03	0.37 ± 0.09	0.04	0.16
Bacteria	0.43 ± 0.07	0.39 ± 0.01	0.43 ± 0.08	0.03	0.87

^1^ Means (± standard error) within a row (overall) followed by different superscript are significantly different, General Linear Model (GLM), *p* < 0.05, least significant difference test. ^2^ D0 = 0 g, D10 = 10 g, D100 = 100 g, levels of supplement BSFLF. ^a^—*p* < 0.05; ^b^—*p* < 0.01 vs. control. For each diet, n = 3.

**Table 8:** Biochemical and morphological parameters of the blood ^1^.

Parameter	Diets ^2^			SEM	*p*-Value
	D0	D10	D100		GLM
	n = 5	n = 5	n = 5		
TP (g/L)	98.13 ± 5.83	90.70 ± 2.73	90.32 ± 4.04	2.39	0.32
ALB (g/L)	32.33 ± 1.43	34.27 ± 0.83	32.88 ± 1.37	0.65	0.47
GLB (g/L)	65.80 ± 7.05	56.43 ± 2.48	57.44 ± 3.63	2.64	0.28
ALB/GLB	0.52 ± 0.08	0.61 ± 0.03	0.58 ± 0.04	0.03	0.41
UREA (mmol/L)	3.08 ± 0.24	4.70 ± 0.22 ^c^	3.41 ± 0.29	0.23	<0.001
CREA (mmol/L)	74.65 ± 5.55	75.38 ± 5.28	70.60 ± 3.22	2.44	0.70
TBIL (µmol/L)	2.44 ± 0.24	3.80 ± 0.75 ^d^	5.79 ± 1.56 ^a^	0.63	0.06
ALT (IE/L)	22.66 ± 2.15	25.16 ± 2.01	24.09 ± 1.56	0.99	0.60
AST (IE/L)	64.65 ± 3.77	66.19 ± 8.89	61.61 ± 5.28	3.19	0.81
ALP (mmol/L)	92.89 ± 23.22	73.48 ± 8.00	93.03 ± 30.36	11.44	0.73
CHOL (mmol/L)	6.16 ± 0.48	7.11 ± 0.66	4.74 ± 0.34 ^a^	0.37	0.01
TRIG (mmol/L)	0.32 ± 0.02	0.33 ± 0.01	0.32 ± 0.00	0.01	0.54
GLU (mmol/L)	2.66 ± 0.27	3.02 ± 0.27	2.20 ± 0.13 ^a^	0.15	0.05
Ca (mmol/L)	2.75 ± 0.13	2.82 ± 0.05	2.74 ± 0.06	0.04	0.73
P (mmol/L)	2.19 ± 0.24	2.02 ± 0.18	2.13 ± 0.15	0.10	0.78
Ca/P	1.68 ± 0.16	1.85 ± 0.16	1.69 ± 0.12	0.08	0.56
Mg (mmol/L)	0.49 ± 0.11	0.49 ± 0.03	0.54 ± 0.07	0.04	0.81
Fe (µmol/L)	24.46 ± 1.66	27.37 ± 1.61	24.84 ± 1.19	0.82	0.29
WBC (10^9^/L)	13.47 ± 0.87	12.48 ± 2.47	13.04 ± 1.48	0.87	0.90
RBC (10^12^/L)	7.50 ± 0.19	7.68 ± 0.30	7.54 ± 0.12	0.11	0.79
HGB (g/L)	94.52 ± 3.68	93.74 ± 5.42	90.14 ± 0.94	1.96	0.64
HCT (%)	37.31 ± 1.14	37.05 ± 2.28	35.88 ± 0.42	0.76	0.73
TAWSA (mg/g)	10.96 ± 0.55	11.59 ± 1.79	12.59 ± 1.00	0.48	0.56

^1^ Means (± standard error) within a row (overall) followed by different superscript are significantly different, General Linear Model (GLM), *p* < 0.05, least significant difference test. ^2^ D0 = 0 g, D10 = 10 g, D100 = 100 g, levels of supplement BSFLF. ^a^—*p* < 0.05; ^c^—*p* < 0.001;^d^—*p* ≤ 0.10. vs. control. For each diet, n = 5.

**Table 9:** Indicators of nonspecific resistance of experimental animals ^1^.

Parameter	Diets ^2^			SEM	*p*-Value
	D0	D10	D100		GLM
	n = 5	n = 5	n = 5		
LA, %	44.11 ± 2.60	60.10 ± 6.02 ^a^	56.71 ± 6.63 ^d^	0.32	0.08
Lysozyme, mkg/mL	0.78 ± 0.04	1.36 ± 0.28 ^a^	1.29 ± 0.33	0.14	0.18
AU/TP	3.10 ± 0.20	4.12 ± 0.38 ^a^	3.97 ± 0.39 ^d^	0.21	0.07
BA, %	43.56 ± 1.69	46.67 ± 2.69	49.33 ± 2.14 ^a^	1.27	0.16
PA, %	58.83 ± 2.09	55.67 ± 5.02	52.60 ± 3.88	2.96	0.99
PI	3.43 ± 0.21	2.78 ± 0.27 ^d^	3.60 ± 0.06	0.14	<0.05
PAM	1.80 ± 0.22	1.46 ± 0.17	1.89 ± 0.12	0.10	0.16

^1^ Means (± standard error) within a row (overall) followed by different superscript are significantly different, General Linear Model (GLM), *p* < 0.05, least significant difference test. ^2^ D0 = 0 g, D10 = 10 g, D100 = 100 g, levels of supplement BSFLF. ^a^—*p* < 0.05; ^d^—*p* ≤ 0.10. vs. control. For each diet, n = 5.

## Data Availability

Not applicable.
